# A Sulfur Copolymers (SDIB)/Polybenzoxazines (PBz) Polymer Blend for Electrospinning of Nanofibers

**DOI:** 10.3390/nano9111526

**Published:** 2019-10-26

**Authors:** Ronaldo P. Parreño, Ying-Ling Liu, Arnel B. Beltran

**Affiliations:** 1 Department of Chemical Engineering, De La Salle University, 2401 Taft Avenue, Manila 1004, Philippines; 2 Chemicals and Energy Division, Industrial Technology Development Institute (ITDI), Department of Science and Technology (DOST), Taguig 1631, Philippines; 3 Department of Chemical Engineering, National Tsing Hua University, Hsinchu 30013, Taiwan; liuyl@mx.nthu.edu.tw; 4 Center for Engineering and Sustainable Development Research, De La Salle University, 2401 Taft Ave, Manila 1004, Philippines

**Keywords:** sulfur copolymers, polybenzoxazines, electrospinning, polymer blend, nanofibers, inverse vulcanization

## Abstract

This study demonstrated the processability of sulfur copolymers (SDIB) into polymer blend with polybenzoxazines (PBz) and their compatibility with the electrospinning process. Synthesis of SDIB was conducted via inverse vulcanization using elemental sulfur (S_8_). Polymer blends produced by simply mixing with varying concentration of SDIB (5 and 10 wt%) and fixed concentration of PBz (10 wt%) exhibited homogeneity and a single-phase structure capable of forming nanofibers. Nanofiber mats were characterized to determine the blending effect on the microstructure and final properties. Fiber diameter increased and exhibited non-uniform, broader fiber diameter distribution with increased SDIB. Microstructures of mats based on SEM images showed the occurrence of partial aggregation and conglutination with each fiber. Incorporation of SDIB were confirmed from EDX which was in agreement with the amount of SDIB relative to the sulfur peak in the spectra. Spectroscopy further confirmed that SDIB did not affect the chemistry of PBz but the presence of special interaction benefited miscibility. Two distinct glass transition temperatures of 97 °C and 280 °C indicated that new material was produced from the blend while the water contact angle of the fibers was reduced from 130° to 82° which became quite hydrophilic. Blending of SDIB with component polymer proved that its processability can be further explored for optimal spinnability of nanofibers for desired applications.

## 1. Introduction

Elemental sulfur (S_8_) is a largely available untapped resource with an estimated amount of more than 60 million tons generated annually from the hydrodesulfurization process of natural gas and oil to reduce sulfur dioxide (SO_2_) emissions from the combustion of these fossil fuels [1,2]. Sulfur as a by-product of the petroleum refining process causes the “excess sulfur problem” because of rising global petroleum demand [2,3]. Sulfur is mostly used for the production of sulfuric acid and phosphates for fertilizers, synthetic rubber and cosmetics but still has limited demand with an estimated surplus of 7 million tons annually [1]. The abundant amount of excess sulfur coupled with its unique properties has created renewed interest in sulfur as an alternative feedstock for sulfur-based materials [4]. Previous studies were carried out to overcome the challenges of processing elemental sulfur into new materials such as reinforcing filler for composites [5], chemically modified sulfur for sustainable construction material [6], and as feedstock for reacting with another polymer to obtain network structured and soluble sulfur-rich copolymers [7]. However, the processability of sulfur still remains the main challenge for its direct utilization.

Early research works have already proven the ability of S_8_ to copolymerize with different polymers such as propylene sulfide [8,9], styrene [10], diynes [11] and cyclic disulfides [12] but the polymeric materials formed have limited processability and tunability of properties [1]. Until recently, polymeric material from direct utilization of sulfur was achieved via a solvent free copolymerization process known as “inverse vulcanization” [1,2]. It is the reversal of established vulcanization process where sulfur served as the backbone of the polymeric material while the carbon aromatic comonomer, 1,3-diisopropenyl benzene (DIB) bind the sulfur chains to form more stable sulfur copolymers (SDIB) [5]. The potential of SDIB as processable polymers with its distinctive properties from the high sulfur content and improved chemical and processing characteristics opened opportunities for more research efforts on potential applications. Sulfur copolymers produced using different crosslinkers via inverse vulcanization were evaluated for mercury capture application [3] while another study used inverse vulcanization in a reaction of sulfur with natural dienes to produce flexible sulfur films [13]. Taking advantage of the good electrochemical properties, SDIB became a new addition to the emerging class of electroactive polymers used as active cathode material for performance improvement of lithium sulfur (Li-S) batteries [14]. This was so far the most promising application of SDIB and it continues to constitute the majority of recent advances in SDIB application [14].

Further advances and breakthroughs in SDIB would require looking at its potential applications in considerations of the polymer composition and processing characteristics. Sulfur copolymers with comonomer ratio of 50 wt% DIB were found to be completely soluble in non-polar organic solvents [1]. They are amorphous in nature and classified as uncrosslinked thermoplastic copolymers with low molar mass and high polydispersity [1,2]. These attractive features provided the processing options that could be explored through this work by trying out diverse fabrication techniques and robust modification methods to produce desired structures and preferred surface properties. One of the commonly used polymer processing methods is polymer blending to exploit the properties of synergy formed with other polymers. This involved mixing two polymers with properties that can be manipulated by the component polymers [15]. It was demonstrated previously that polymer blend of SDIB with component polymer showed compatibility in electrospinning process to produce electrospun fiber mats [16]. This study is, to the best of our knowledge, the only successful polymer blending that has used SDIB from inverse vulcanization to further process it into nanofiber via electrospinning technique. Electrospinning as promising fabrication method have shown beneficial effects on property enhancement brought about by the morphology of the nanofibers [17]. It is considered to be a versatile process for producing the desired fiber structures, porosity, orientations and dimensions [18] because of its controllable parameters such as solution, process and ambient conditions [19,20,21]. Moreover, electrospinning can be applied for different types of materials such as natural and synthetic polymers, polymer blends, composite with metal or ceramic particles, and nanocomposites [20]. Because of these merits, electrospinning became known for producing high-surface area materials and recognized as the method that can provide wide range of applications, including catalysis [22], energy storage [23], biosensing [24], drug delivery [25] and membranes [26]. Hence, SDIB as a component polymer in the blending process has great potential for delivering enhanced materials through the application of electrospinning.

In this study, we focus on using sulfur copolymers synthesized via inverse vulcanization of S_8_ to investigate its processability with a polymer given its processing characteristics and structure. Polybenzoxazines (PBz), a thermoset material, was selected as component polymer because of its unique properties such as chemical inertness, high thermal stability, flexural strength, as well as near zero volumetric change upon curing [27,28,29]. Another advantage of PBz is the easy thermal curing without hardeners or catalysts resulting to high mechanical and thermal properties [28,29]. This is due to crosslinking of PBz that occurs via thermally activated ring-opening polymerization (ROP) of the oxazine ring in the main polymer chain [28,30]. These remarkable properties led to exploring the potential synergy created by polymer blending of PBz with SDIB. Herein, the combination of SDIB with thermoset material PBz processed into polymer blends were examined for the physical behavior and phase structure prior to subsequent processing. Further processing of the polymer blend was conducted to determine what material can be produced when its compatibility to form nanofibers was investigated. The versatility of electrospinning allowed the control of process parameters that govern the spinning process to accommodate the polymer properties specific for SDIB/PBz as polymer blend. Electrospun nanofiber mats were characterized to evaluate the effect of copolymers-polymer blending on resulting fiber morphology and structure, thermal and structural properties, surface property and solubility. This work offered a different insight on process option that could be further studied to find new applications of SDIB as an emerging class of polymeric material. The main contribution of this study is to demonstrate that processability of SDIB as component in polymer blend and its spinnability to produce electrospun nanofibers via electrospinning has the potential for further optimization of the desired properties of nanofibers.

## 2. Materials and Methods

### 2.1. Materials

Sulfur (S_8_) (powder, sublimed, Alfa Aesar 99.5%), 1,3-diisopropenylbenzene (DIB) (TCI Chemicals, >97.0%) were used as received without any purification in the synthesis of sulfur copolymers. Polybenzoxazines (PBz) was prepared in the lab as reported in the work of Liu et al. [30]. Dimethylsulfoxide (DMSO) (ACS grade, Echo, 99.9%), and Tetrahydrofuran (THF) (inhibitor free high purity, Tedia, 99.8%) were used as received without any purification.

### 2.2. Synthesis of SDIB via Inverse Vulcanization Process

The synthesis of sulfur copolymers (SDIB) was undertaken using the procedure described in the work of Chung et al. [1]. The comonomer ratio of sulfur to DIB used in the synthesis was 50 wt%. Synthesis of SDIB was undertaken by adding the sulfur powder in a 25 mL glass vial equipped with a magnetic stir bar and then, heated to 185 °C in a thermostated oil bath for 5 min until a clear orange colored molten phase was formed. DIB was then directly added to the molten sulfur in the glass vial with the molten sulfur. Then the resulting mixture was continuously stirred for 8–10 min at 185 °C until vitrification was complete. Lastly, the product from the reaction after complete vitrification of the solution was cooled to room temperature.

### 2.3. Preparation of Polymer Blend

Polymer blending process of SDIB and PBz was carried out using simple mixing technique by dissolving the polymers in separate mixture of solvents. Three polymer blends of SDIB and PBz were prepared according to concentration of PBz (fixed at 10 wt%) and SDIB (0, 5 and 10 wt%) in the polymer solution with final blends coded as SDIB/PBz-Y where Y (0, 5 and 10) denotes the varying compositions of component polymer SDIB. For SDIB/PBz-0 or pure PBz, electrospinning solution was previously prepared in a mixture of DMSO/THF with a ratio of 1:3 (*v*/*v*) which was used as basis for blending of SDIB/PBz-5 and SDIB/PBz-10. Initially, two separate solutions of SDIB and PBz in DMSO/THF were prepared. After continuous stirring of the two solutions for at least 24 h at room temperature when both SDIB and PBz were fully dissolved, the two solutions were mixed in one solution. Then, the blend of two solutions was stirred for at least 1 h at room temperature to fully mix the polymers into one another to form the final polymer blend.

### 2.4. Electrospinning of Polymer Blend

The electrospun nanofiber mat from the polymer blend was produced using vertically-aligned electrospinning apparatus composed of a 10 mL syringe with needle (ID = 0.8 mm) connected to a syringe pump, a ground electrode, and a high voltage supply (Falco Enterprise Co., Ltd., Taipei, Taiwan). Three electrospun nanofibers having polymer concentration of 0, 5 and 10 wt% SDIB were prepared and coded as electrospun (ES) nanofibers of SDIB and PBz (SDIB/PBz) or ES-SDIB/PBz-X, where X (0, 5 and 10) identifies the amount of SDIB while PBz was fixed to polymer concentration of 10 wt%. The needle was connected to the high voltage supply, which generates positive DC voltages up to 40 kV. Polymer blend of SDIB/PBz was placed in a 10 mL syringe which was ejected through a needle spinneret by a syringe pump (Falco Enterprise Co.) with a mass flow rate of 1.00 mL h^−1^ and tip-to-collector distance (TCD) of 15 cm. Applied voltage was varied from 10 kV to 20 kV in the electrospinning process depending on the change in concentrations of polymer blends (0, 5 and 10 wt% SDIB concentrations) while other process parameters were not changed. Electrospinning process was carried out at ambient conditions. After electrospinning, the electrospun nanofibers were set aside for at least 24 h to vaporize remaining solvent prior to thermal treatment. For pure PBz, the curing was conducted at temperatures of 80 °C, 100 °C, 160 °C, 200 °C and 240 °C, each for 1 h inside the oven (Deng Yng, DH 400) to thermally crosslinked the PBz. Then, thermally treated mats were cool down to room temperature inside the oven and then, removed from the collector plate. For the electrospun nanofibers, ES-SDIB/PBz-5 and ES-SDIB/PBz-10, the thermal treatment was initially tested based on the curing conditions used for ES-SDIB/PBz-0 and adjusted to new thermal curing conditions using only two-step temperature of 160 °C and 240 °C each for 1 h.

### 2.5. Characterization of Electrospun Nanofiber Mat

Characterization of electrospun nanofibers mats were conducted to evaluate the resulting properties from blending of SDIB with PBz and to understand the effect of the physical structure and behavior of blend on the spinnability of blend for nanofiber formation. Fourier-transform infrared spectroscopy (FTIR) spectra were obtained using a Perkin Elmer Spectrometer at the wavenumber between 400 cm^−1^ to 4000 cm^−1^. SEM-EDX analysis was carried out with a Scanning Electron Microscope (SEM) (FEI Helioz Nanolab 600i, Eindhoven, The Netherlands) with Energy Dispersive X-ray Spectroscopy (EDX) (Oxford Instrument X-Max, Abingdon, UK.) at the Advanced Device and Materials Testing Laboratory (ADMATEL) of DOST. Thermal property was measured by differential scanning calorimetry (DSC) using a Q20, TA instrument at heating rate of 10 °C/min in the temperature range of 50–450 °C under nitrogen environment. Water contact angle (WCA) was measured using an instrument from First Ten Angstroms (FTA, Model: FTA 1000 B) to evaluate the surface properties of the electrospun nanofiber mats. The contact angle data were obtained from the average of three replicates from five measurements of samples. Solubility tests of the electrospun nanofibers were undertaken by soaking the samples for at least 24 h in common solvents to determine its solvent resistance after SDIB was incorporated.

## 3. Results and Discussion

### 3.1. Preparation of Polymer Blend

**Figure 1:**
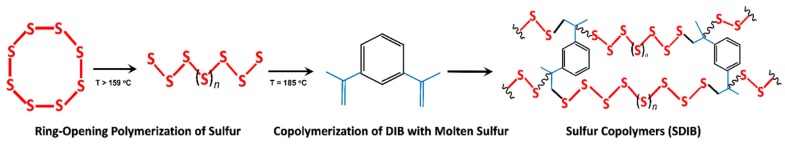
Schematic representation of sulfur copolymers (SDIB) produced via inverse vulcanization of S_8_ with 1,3-diisopropenyl benzene (DIB).

The SDIB used for blending process was synthesized via inverse vulcanization of S_8_ with comonomer DIB as shown in Figure 1. SDIB has polysulfide backbone with aromatic carbon monomers bound to sulfur chains to form stable copolymers.

The processability of SDIB in polymer blending process was conducted using the common and simple technique of dissolving the polymers in common mixture of solvents. It is known that blending of polymers having each individual characteristic is considered an effective technique to develop new material with desired properties [31,32]. However, employing the appropriate method is critical in achieving a homogeneous system of polymer mixtures. Moreover, polymer blending process are primarily influenced by the nature of the copolymers-polymer mixture and the ratio of the component polymers which significantly affect the behavior and phase structure of the blends [33]. In mixing SDIB and PBz which represents copolymers-polymer system with different amount of SDIB, different approaches of blending were carried out initially to find out the appropriate strategy to produce homogenous blend instead of formation of gel. It is important to obtain a miscible blend as property mix depends on the degree of miscibility of the two polymers at molecular level [33,34].

**Figure 2:**
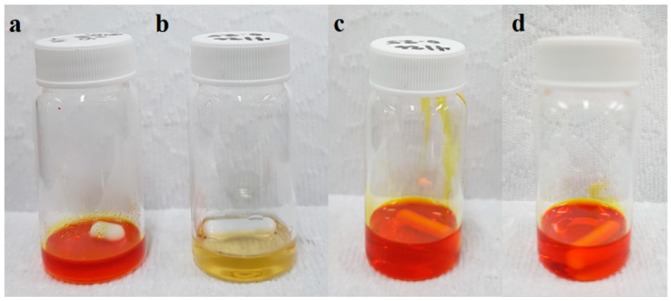
Polymer blend preparation from: (**a**) SDIB solution; (**b**) PBz solution; (**c**) Polymer Blend of SDIB/PBz (5/10 wt%); and (**d**) Polymer blend of SDIB/PBz (10/10 wt%).

In this work, blending was achieved by dissolving separately the PBz and SDIB in separate mixtures of DMSO/THF solvent to form two polymer solutions and later on mixed together into the final polymer blend. This approach resulted to miscible blends with SDIB and PBz mixing in the solutions to produce homogeneous, transparent and single-phase structure as shown in Figure 2. As observed in the polymer blend, the homogeneity was very evident with the absence of separation of interface when two phases appear in the blend [15,35].

**Figure 3:**
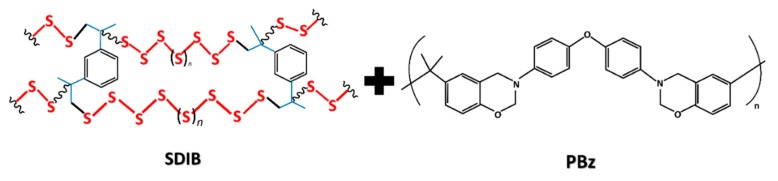
Polymer blending of SDIB and PBz with chemically aromatic structures.

Mixing of polymers results in a miscible blend due to the special interactions that exist between the component polymers which could be due to similarity in structure [36]. In this case, the miscibility of SDIB and PBz solutions indicated the presence of special interaction between the copolymers-polymer components. This interaction could be attributed to the nature of the copolymers, SDIB and component polymer, PBz which both are chemically aromatic in their structures. The chemical structures of SDIB and PBz used in the blending process both having aromatic structures are shown in Figure 3. Polymers with the presence of phenyl groups tend to have special attractions and associate with each other during mixing allowing the polymers to mix and formed miscible blends [33].

Another important consideration for mixing polymers is the amount of components in the polymer blend. A lower amount of one polymer compared to the other component promotes a discontinuous phase in the polymer blend while a continuous phase occurs when there is an equal amount of the two polymers in the polymer blend [33]. For SDIB/PBz-10, the equal amount of polymer also contributed to a stronger interaction between SDIB and PBz because of the formation of continuous phases in the two polymers which resulted to miscible blend. For unequal amounts of polymer blend SDIB/PBz-5, the miscibility of the polymer blend was not affected, exhibiting a similar homogeneous system. The interaction present due to the nature of the two polymers could be responsible for the degree of miscibility from the minimal change in the amount of SDIB in the polymer blend which also produced a miscible blend. The miscibility and single-phase structure of the SDIB/PBz polymer blend were integral in the compatibility on electrospinning.

### 3.2. Electrospinning of Polymer Blend

In the polymer blend, the amount of polymer components of SDIB (0, 5 and 10 wt%) and PBz (10 wt%) represents the polymer concentration which have direct effects on spinnability. Polymer concentration is considered as a primary parameter of the electrospinning process which is the main consideration for other process parameters. For SDIB/PBz polymer blends with varying concentrations of components, spinning process was govern by adjusting only the applied voltage to accommodate the change in polymer concentration while other parameters such as flow rate and TCD were not changed. Electrospinning of SDIB/PBz-0 was conducted with an applied voltage of 10 kV that produced nanofibers. First run of SDIB/PBz-5 polymer blend was conducted at the same applied voltage of 10 kV but showed unsatisfying results with polymer dopes accumulating at the needle tip which produced mostly electrosprayed droplets instead of fibers. The polymer concentration effect of the polymer blend is directly related to the viscosity and surface tension to obtain nanofibers [20]. By applying high voltage in electrospinning process, the polymer solution gets charged and Taylor cone is formed [19]. However, when the Taylor cone is applied with sufficient strength of electric field which overcomes the surface tension of the droplet and polymer solution feed at the tip of needle becomes electrically charged, the charged polymer solution is ejected as a jet to form nanofibers [37]. Addition of SDIB in the polymer concentration caused insufficient applied voltage which was not enough to overcome the surface tension. This led to disruptions of jet formation due to weaker electrically charged polymer solution. In subsequent runs, the spinnability of polymer blend to form nanofibers was achieved with higher applied voltage. It was realized at this voltage level through the formation of nanofiber from the SDIB/PBz blend but optimization with other process parameters is still needed to obtain desired morphology and properties of nanofibers. The applied voltage used for the polymer blends to obtain nanofibers are shown in Table 1.

**Figure 4:**
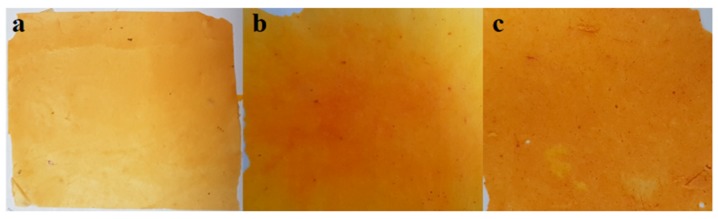
Electrospun Nanofibers of: (**a**) Electrospun SDIB/PBz with 0 % SDIB (ES-SDIB/PBz-0); (**b**) ES-SDIB/PBz-5; and (**c**) ES-SDIB/PBz-10.

The electrospun nanofiber mats produced from polymer blend were thermally cured to remove residual solvent and enhance mechanical and thermal properties from the PBz component polymer. The thermally cured mats of SDIB/PBz are shown in Figure 4. An observable difference in physical appearance was evident in the surface and color of the nanofiber mats which could be directly related to the SDIB added. ES-SDIB/PBz-0 has smoother and uniformed colored light brown surface while for ES-SDIB/PBz-5 and ES-SDIB/PBz-10, a similar smooth surface was observed but with some presence of areas with uneven surface that has darker shades of yellowish brown. The color of the nanofiber mats with the addition of SDIB changed from light brown to light yellowish brown for ES-SDIB/PBz-5 and darker yellowish brown for ES-SDIB/PBz-10. The changed in surface and uneven color tone was attributed to the presence of sulfur in the surface of the nanofibers.

### 3.3. SEM-EDX of Electrospun Nanofibers

**Figure 5:**
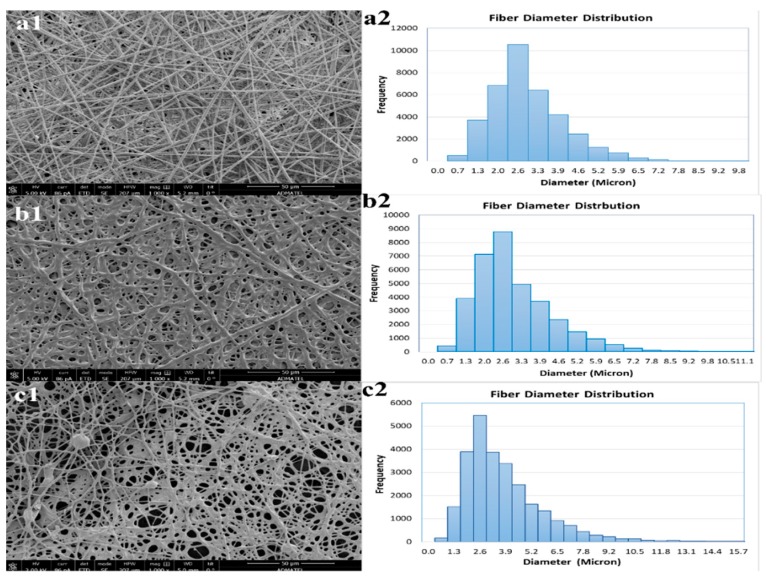
Fiber diameter distribution based on SEM micrographs of: (**a1**,**a2**) ES-SDIB/PBz-0; (**b1**,**b2**) ES-SDIB/PBz-5; and (**c1**,**c2**) ES-SDIB/PBz-10 (Magnification: 1000× and scale bar: 50 µm).

The SEM images were used to evaluate the morphology of the electrospun nanofibers from the polymer blending of SDIB with PBz. For ES-SDIB/PBz-0, analysis of SEM image in Figure 5(a1) showed a uniform fiber morphology with an average fiber diameter of 2.668 ± 0.987 µm and an evenly distributed fiber diameter between 1.3–3.3 µm (Figure 5(a2)). Comparing the ES-SDIB/PBz-5 in Figure 5(b1,b2), the average fiber diameter of 2.578 ± 1.041 µm was almost the same with ES-SDIB/PBz-0 but the fiber diameter was slightly non-uniform as indicated by the skewed distribution. This means that more fibers have diameter less than the average fiber diameter and have broader dispersion concentrated between 1.3–4.6 µm. The increase in concentration from SDIB (5 wt%) in the polymer blend had no significant effect on the fiber diameter but a more broader fiber distribution. In the case of ES-SDIB/PBz-10 as shown in Figure 5(c1,c2), the fiber diameter slightly increased by 0.5 um with average fiber diameter of 3.073 ± 1.350 µm resulting to more dispersed distribution between 1.3–6.5 µm indicating increased non-uniformity in fiber diameter as compared to both ES-SDIB/PBz-0 and ES-SDIB/PBz-5. These results were attributed to the increasing SDIB concentration which influenced directly the polymer concentration resulting to increased surface tension of the polymer mixture. Higher applied voltage was required to overcome the increased in surface tension to produce interactions between electrically charged polymer blend and external electric field to reach Taylor cone formation. However, the higher electric field from applied voltage resulted in increased fiber diameter greater than the desired size for nanofibers which are classified as microfibers as well as a change in fiber morphology. The electrospinning process can be further optimized for SDIB/PBz blend by adjusting other process parameters aside from voltage to produce the desired nanofiber size and morphology.

**Figure 6:**
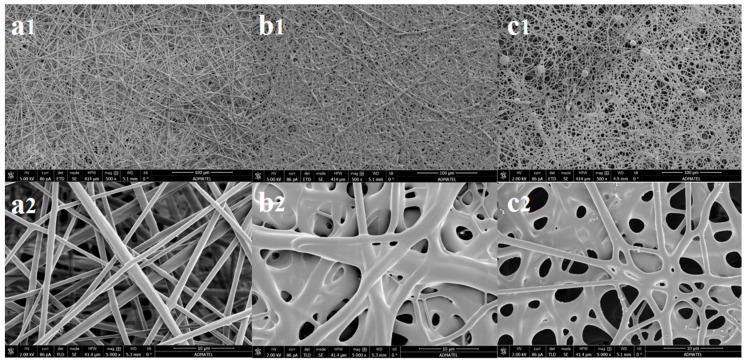
SEM micrographs of electrospun nanofibers of (**a1**,**a2**) ES-SDIB/PBz-0; (**b1**,**b2**) ES-SDIB/PBz-5; and (**c1**,**c2**) ES-SDIB/PBz-10; (Magnification for **a1**, **b1** and **c1**: 500×; **a2**, **b2** and **c2**: 5000×; scale bar: 10 and 100 µm).

Further analysis of SEM images of all electrospun nanofibers in Figure 6(a1–c2) exhibited microstructures of randomly-oriented continuous, interconnected nanofibrous structures. ES-SDIB/PBz-0 in Figure 6(a1,a2) showed fibrous structure with highly uniform and smooth fibers with no occurrence of beads. Formation of randomly-oriented continuous nanofibers were also seen in both ES-SDIB/PBz-5 and ES-SDIB/PBz-10 in Figure 6(b1–c2) but not as uniform and as smooth as ES-SDIB/PBz-0. Based on these results, the polymer blending allowed the spinnability of SDIB/PBz by attaining the required molar mass to produce nanofibers. Previous study on electrospinning of pure sulfur copolymers resulted in unsatisfying results which produced broad fiber diameter with heavy beads and melting of fibers at the contact points [16]. It was explained that a polymer below the required molar mass may be blended with another polymer to produce a homogeneous blend capable of forming nanofibers [38]. SDIB has low molar mass with number-average molecular weight (*Mn*) of 1262 g-mol^−1^ [2] while PBz has high molar mass with *Mn* of 8000–9000 g-mol^−1^ [28]. It was found out that, generally, higher molecular weight of the polymer results in desirable viscosity aside from the effect of concentration for fiber formation [20]. Although nanofibers were formed, the presence of few beads-on-string in ES-SDIB/PBz-10 were observed which could be due to the high voltage application. Increasing the applied voltage in electrospinning causes changes in the morphology and structure of the fibers [19] and results in the formation of beads [39]. Another observable change in the microstructures of the ES-SDIB-5 and ES-SDIB/PBz-10 were the occurrence of partial aggregation and conglutination with each fibers (Figure 6(b2,c2)). The fine tuning of nanofiber morphology and structure by increasing the amount of SDIB in the polymer blend could be manipulated by adjusting other parameters such as feed rate, TCD and needle size to suit the polymer concentration.

**Figure 7:**
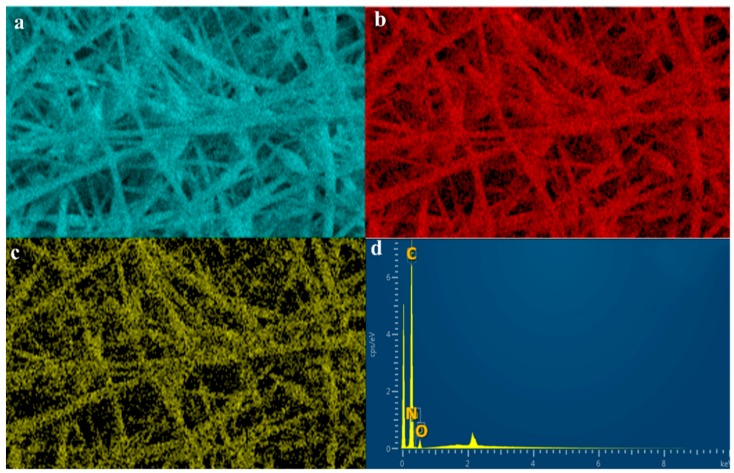
Energy Dispersive X-ray Spectroscopy (EDX) map and spectra of ES-SDIB/PBz-0 showing the elemental compositions: (**a**) carbon; (**b**) oxygen; (**c**) nitrogen and (**d**) spectra without sulfur (S) peak; (scale bar = 5 µm); Other peaks without label in the spectra are Au used as coating material.

**Figure 8:**
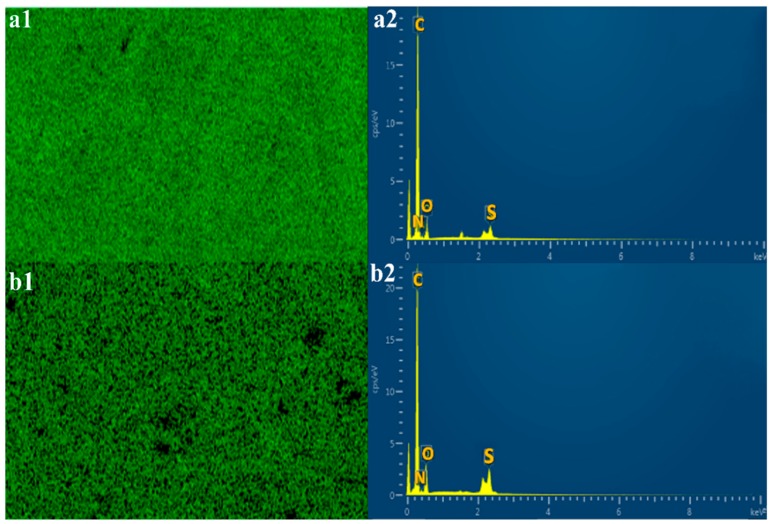
EDX Map and spectra of (**a1**,**a2**) ES-SDIB/PBz-5 with the presence of sulfur (S) (green color) and sulfur peak; and (**b1**,**b2**) ES-SDIB/PBz-10 showing the presence of sulfur (S) (dark green color) and sulfur peak; (scale bar = 5 µm); Other peaks without label in the spectra are Au used as coating material.

The EDX spectra and map were analyzed to determine the overall chemical composition and the distribution of the chemical elements of interest in the electrospun nanofibers. As observed in the EDX map and spectra of ES-SDIB/PBz-0 in Figure 7a–d, as expected, sulfur was not present in the nanofibers where a carbon element predominantly appeared in blue color with oxygen and nitrogen while the EDX spectra showed only peaks of carbon, nitrogen and oxygen. The presence of sulfur (green color) in the EDX map of ES-SDIB/PBz-5 and ES-SDIB/PBz-10 in Figure 8(a1–b2) confirmed the incorporation of SDIB in the nanofibers. Based on the EDX spectra for both electrospun nanofibers with SDIB, sulfur peaks were observed alongside with peaks assigned to carbon, nitrogen and oxygen. In addition, the relative peak heights of the chemical elements present in the polymer blends were clearly correlated with the respective amount present in the nanofibers. Applying this to the sulfur peak observed in the EDX spectra, the relatively shorter sulfur peak in the nanofibers with lower concentration of SDIB (5 wt%) compared to the sulfur peak with higher concentration of SDIB (10 wt%) showed the quantitative amount of SDIB in the two electrospun nanofibers. This confirmed that greater concentration of S was present in the nanofibers with 10 wt% SDIB (darker green color) as compared to the nanofibers with 5 wt% SDIB (lighter green color).

### 3.4. FTIR Spectroscopy

**Figure 9:**
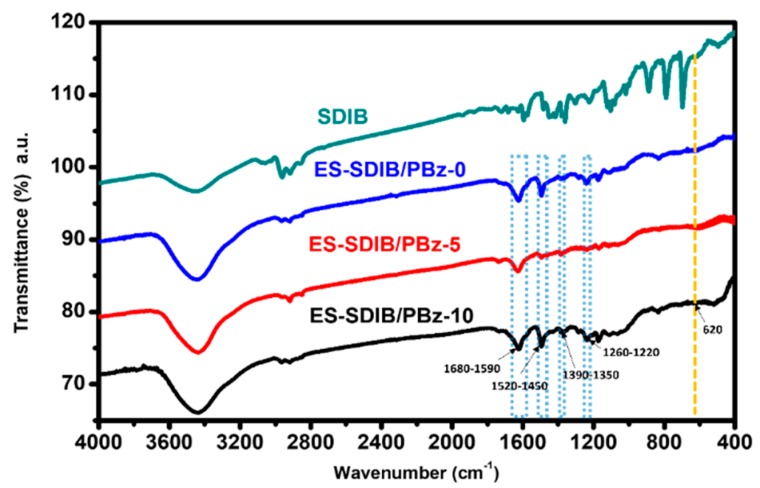
Fourier transform infrared (FTIR) spectra of pure SDIB and ES-SDIB/PBz (0, 5 and 10) nanofibers.

To confirm the effect of blending of SDIB, electrospun nanofibers were characterized by using Fourier transform infrared (FTIR). The FTIR spectra of ES-SDIB/PBz-5 and ES-SDIB/PBz-10 were compared to the absorption characteristic bands of unblended SDIB and ES-SDIB/PBz-0 as shown in Figure 9. The spectrum of ES-SDIB/PBz nanofibers contained all the characterization absorption bands of ES-SDIB/PBz-0 assigned to benzoxazine structure at 1260–1220 cm^−1^ due to asymmetric stretching of C-O-C bonds, 1390–1350 cm^−1^ due to CH_2_ wagging and at 1520–1450 cm^−1^ for the tri-substituted benzene ring as well as C=C bond at 1680–1590 cm^−1^. The spectra of electrospun nanofibers with SDIB showed no difference to that of electrospun PBz without SDIB. There was no absorption band associated with C-S bond at 620 cm^−1^ (dashed yellow line in Figure 9) that would indicate the effect of SDIB in the structural group of the electrospun nanofibers. This confirmed that the SDIB did not have any effect on the chemistry of PBz and no specific interactions occurred during the polymer blending. Moreover, SDIB and PBz did not undergo any chemical reaction during the blending process which is desirable for blending of materials.

### 3.5. Thermal Property

**Figure 10:**
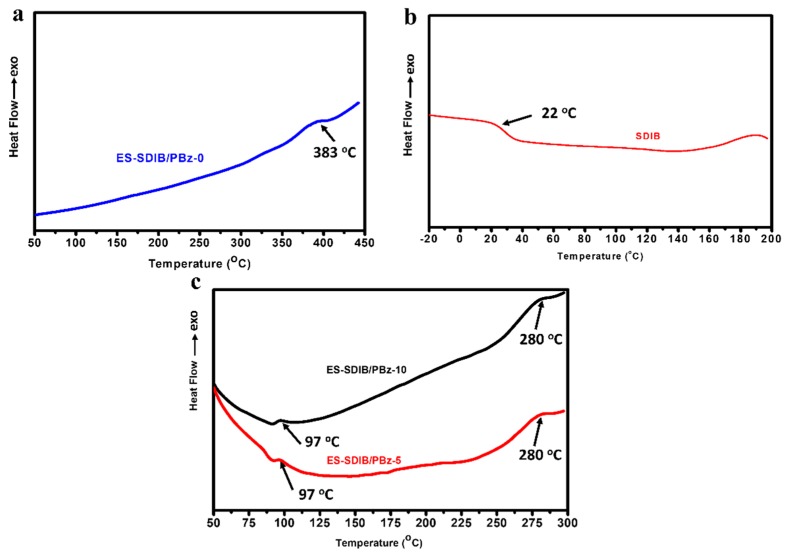
Differential scanning calorimetry (DSC) curves of (**a**) ES-SDIB/PBz-0 (pure PBz); (**b**) pure SDIB; and (**c**) ES-SDIB/PBz-5 and ES-SDIB/PBz-10 with the new glass transition temperatures.

Pure PBz polymer exhibits a high glass transition temperature (*Tg*) [40] while SDIB (10–50 wt% S ratio) has a very low glass transition temperature that ranged from −14 °C to 28 °C [1]. Based on the DSC measurements as shown in Figure 10a,b, the electrospun nanofibers of pure PBz has a *Tg* of 383 °C while pure SDIB has a *Tg* of 22 °C. Both ES- SDIB/PBz-5 and ES-SDIB/PBz-10 exhibited two new *Tg*’s of 97 °C and 280 °C (Figure 10c) which are between the *Tg* values (22–383 °C) of each component polymers. This result indicated that the degree of miscibility of the polymer blend during electrospinning process changed after formation of fibers made of blend of two polymers. A polymer blend would exhibit two distinct *Tg*’s when the polymer blend is partially miscible where the *Tg* value of each polymer component was affected by the other one [36]. This was evident with distinctly different *Tg*’s but between the values of the pure polymers which showed the effect of interaction of SDIB and PBz in the blend when formed into nanofibers. The same *Tg*’s for both polymer concentrations of 5 wt% and 10 wt% SDIB showed that the amount of SDIB have no significant effect on the thermal property of the electrospun nanofibers. Although based on observation of the polymer blend a high degree of miscibility is observed prior to electrospinning due to the nature of the copolymers-polymer interaction, the application of the electric field during electrospinning may have caused repulsive forces on the surface’s jet of the polymer blend resulting the occurrence of phase-separation for the blend of SDIB and PBz while fibers were formed. The special interactions that existed in the miscible blend of SDIB and PBz were not sufficient to retain miscibility during the electrospinning process. For polymer blends, miscibility is often attained with the occurrence of attractive specific interactions between the component polymers [15]. The new *Tg*’s of the electrospun SDIB/PBz are lower than the PBz polymer but higher than SDIB copolymers which indicate that a new material with properties different from SDIB and PBz was produced.

### 3.6. Water Contact Angle

**Figure 11:**
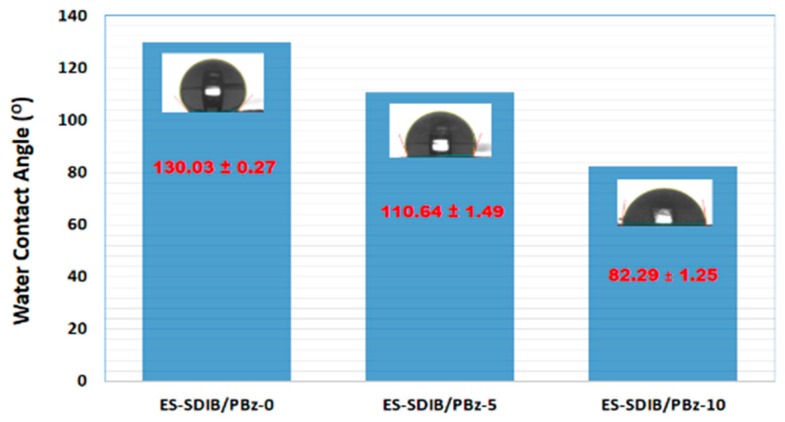
Water contact angles of ES-SDIB/PBz-0, ES-SDIB/PBz-5 and ES-SDIB/PBz-10. Values plotted are means ± standard deviations with three replicates taken per data point.

The effect of blending SDIB with PBz on the hydrophobicity of the electrospun nanofibers’ surface was determined by measuring the water contact angle of the sample nanofibers. Pure PBz exhibited a water contact angle of 130.03 ± 0.27° as shown in Figure 11. The addition of SDIB in the electrospun nanofibers enhanced the hydrophilicity which is inversely correlated with SDIB composition. At concentration of 5 wt% SDIB, the water contact angle was 110.64 ± 1.49° while at concentration of 10 wt% SDIB, the electrospun nanofibers became quite hydrophilic with a water contact angle of 82.29 ± 1.25°. This reduction in water contact angle of the electrospun nanofibers provided surface properties modification which could presumably be caused by the extra hydrogen bonding between the water and the hydroxyl groups from the addition of SDIB.

### 3.7. Solubility

Pure electrospun PBz is insoluble in most commonly used organic solvents such as dimethlyformamide (DMF), dimethyl sulfoxide (DMSO), dimethylacetamide (DMAc) and ethanol (EtOH). The solubility of electrospun nanofibers with SDIB were tested in the same set of common solvents to determine the retention of solvent resistance of the nanofibers with the incorporation of SDIB to PBz. The results in Table 2 showed that electrospun nanofibers were all insoluble in the DMF, DMSO, DMAc and ethanol. This is another way of confirming that SDIB did not have any effect on the chemical structure of the PBz after blending and electrospinning process.

## 4. Conclusions

This work has demonstrated the processability of sulfur copolymers from inverse vulcanization of elemental sulfur blended with polybenzoxazines via electrospinning to produce electrospun nanofibers. Miscible polymer blend was attained by simple mixing of the component polymers which could be mostly attributed to the copolymers-polymer nature resulting from the interactions between the SDIB and PBz. The polymer blends have shown their compatibility to electrospinning and their capability of producing nanofibers with the resulting fiber structure and morphology. However, the effect of polymer properties and composition of SDIB/PBz could be studied further to optimize process conditions of electrospinning for the desired nanofiber morphology and microstructures. The control of other process parameters, particularly the feed rate, could be explored in electrospinning of SDIB. Effective incorporation of SDIB in polymer blend produced a new material which also modified the surface properties but was able to retain some of the desirable properties from the synergy created by blending polymers. Increasing the amount of SDIB in polymer blend could be studied for the resulting property mix of polymer blend using other component polymers. This new processing option for SDIB was able to produce new material but additional research on the optimized amount of SDIB as well as the process conditions of electrospinning process is needed to improve the properties and structures of nanofibers. Nanofibers are important one-dimensional (1D) nanostructures having unique functional properties such as high specific surface areas, porosities, aspect ratio and better pore connectivity. They could have potential applications in adsorption and separation processes as well as in fuel cell membranes.

## Figures and Tables

**Table 1:** Variation in Applied Voltage used for Electrospinning of SDIB/PBz.

Polymer Blend	Applied Voltage, kV					
	10	12	14	16	18	20
SDIB/PBz-0	⁄ ^1^					
SDIB/PBz-5	x ^2^	x	x	⁄		
SDIB/PBz-10	x	x	x	x	x	⁄

^1^ fibers were formed; ^2^ fibers were not formed.

**Table 2:** Solubility of Electrospun SDIB/PBz Nanofibers in Common Solvents.

Sample	Solvent	Solubility
ES-SDIB/PBz-0	DMF, DMSO, DMAc, Ethanol	Insoluble
ES-SDIB/PBz-5	DMF, DMSO, DMAc, Ethanol	Insoluble
ES-SDIB/PBz-10	DMF, DMSO, DMAc, Ethanol	Insoluble
